# From Biocontrol to Synthesis: Innovative Progress of *Paenibacillus* in Mechanism Analysis, Gene Editing and Platform Construction

**DOI:** 10.3390/ijms262210886

**Published:** 2025-11-10

**Authors:** Panhong Yuan, Linjiang Zhu, Zonghui Song, Yasi Wang, Xiaolong Chen

**Affiliations:** College of Biotechnology and Bioengineering, Zhejiang University of Technology, Hangzhou 310014, China

**Keywords:** *Paenibacillus*, biocontrol mechanisms, antibacterial metabolites, gene editing, synthetic biology, industrial biotechnology

## Abstract

*Paenibacillus*, a plant-growth-promoting rhizobacterium, exhibits broad-spectrum biocontrol activity through the production of diverse antibacterial metabolites, competitive niche colonization, induction of systemic resistance, and enhancement of nutrient uptake. The review summarizes recent advances in elucidating the synergistic interactions among its biocontrol mechanisms and their responses to environmental factors. Subsequently, it outlines gene editing and regulatory technologies applicable to *Paenibacillus*. Next, key synthetic biology strategies employed to enhance biosynthetic capabilities are examined. Finally, future prospects and challenges associated with advancing *Paenibacillus* toward precision engineering and high-efficiency applications are discussed. Notably, its role in industrial biotechnology—particularly in the scalable production of industrial enzymes and high-value chemicals—is increasingly recognized as a focal point of growing scientific and commercial interest.

## 1. Introduction

*Paenibacillus*, a plant growth-promoting rhizobacterium endowed with broad-spectrum biocontrol activity and multiple beneficial traits, exhibits considerable application potential in the field of microbial resources [1]. *Paenibacillus* not only synthesizes diverse antibacterial metabolites that effectively inhibit the growth and reproduction of plant pathogens, but also confers comprehensive biological protection and growth enhancement to crops through multiple mechanisms, including ecological niche competition, induction of systemic resistance, and facilitation of nutrient uptake and developmental processes [2]. Furthermore, *Paenibacillus* demonstrates promising applications in pharmaceutical development—particularly in novel antibiotic discovery—and in industrial biotechnology, such as the production of enzymes and biosurfactants [3]. In-depth investigation into its biological characteristics and mode of action is crucial for advancing the development of next-generation biopesticides and biofertilizers, promoting sustainable agricultural practices, and enabling the diversified exploitation of microbial resources.

Research on the biocontrol mechanisms of *Paenibacillus* began earlier in international studies, leading to substantial progress in understanding the biosynthesis and regulation of antimicrobial compounds, ecological competition, and induced systemic resistance [4]. Notably, researchers have systematically characterized the biosynthetic gene cluster responsible for polymyxin production and elucidated its molecular mechanism of action, particularly its ability to disrupt bacterial cell membranes [5]. Leveraging advanced technologies such as CRISPR (Clustered Regularly Interspaced Short Palindromic Repeats)-based gene editing and metabolomics, scientists have further uncovered the functional basis of its biocontrol activity [6]. Nevertheless, the synergistic interplay among these mechanisms remains incompletely understood and therefore warrants further investigation.

In the domain of gene editing, pioneering work has led to the development of CRISPR/Cas9-derived tools tailored for *Paenibacillus*, enabling precise genome editing and dynamic regulatory control in *Paenibacillus* [7]. Subsequent innovations, including temperature-sensitive suicide plasmid systems and marker-free (scarless) genome editing techniques, have been developed to enhance editing efficiency and fidelity [8]. In metabolic engineering, targeted knockout or overexpression of key functional genes has successfully redirected metabolic fluxes, significantly improving the yield of desired bioproducts [9]. Despite these advances, challenges persist, including Cas9-associated cytotoxicity and suboptimal transformation efficiency, which hinder broader implementation.

With regard to synthetic biology platform development, significant enhancements in heterologous product synthesis capacity have been achieved through strategies such as modular pathway design, dynamic regulatory circuits, and chassis strain optimization [10]. Efficient promoter libraries and ribosome binding site (RBS) optimization systems have been established to enable fine-tuned gene expression [11]. Progress has also been made in genome minimization and secretion pathway engineering, contributing to improved strain performance [12]. However, current platforms still face limitations in stability, scalability, and cross-strain compatibility [13]. The efficient assembly of complex biosynthetic pathways and the realization of plug-and-play functionality across different hosts represent critical technical hurdles that must be overcome.

As a pivotal biocontrol agent, research on *Paenibacillus* spans from fundamental biological mechanisms to translational applications. This review first summarizes recent advances in understanding the synergistic interactions among its multifaceted biocontrol mechanisms and their responses to environmental variables. Second, we outline state-of-the-art gene editing and regulatory technologies applicable to this organism within the *Paenibacillus*. Third, we examine key synthetic biology strategies employed to enhance biosynthetic capabilities. Finally, we discuss future prospects and challenges in advancing *Paenibacillus* toward precision engineering and high-efficiency applications. Notably, the role of *Paenibacillus* in industrial biotechnology—particularly in the scalable production of industrial enzymes and high-value chemicals—is emerging as a focal point of increasing scientific and commercial interest.

## 2. Analysis of the Biocontrol Mechanism of *Paenibacillus*

### 2.1. Synthesis and Regulation of Antibacterial Active Substances

**Figure 1:**
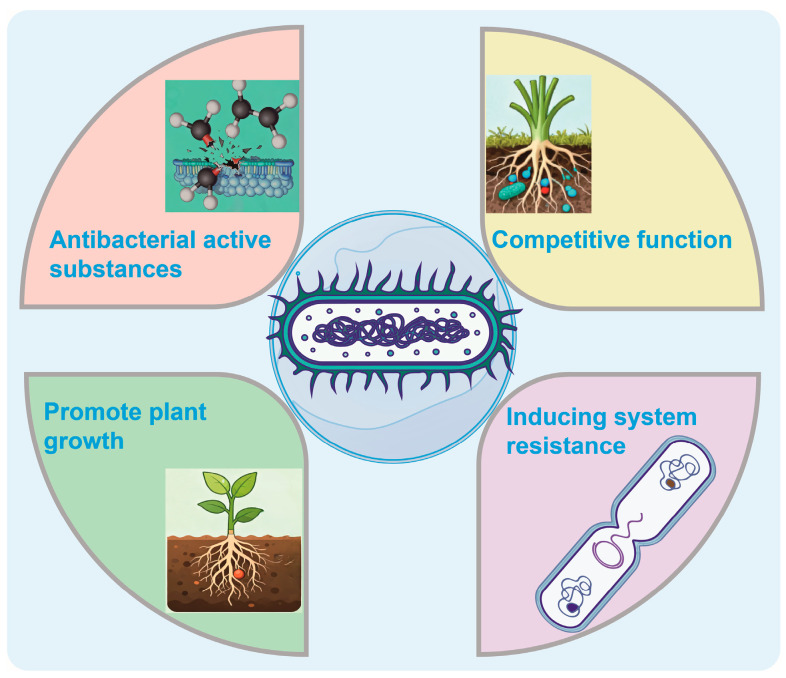
Schematic illustration of the biocontrol mechanism of *Paenibacillus*, including the synthesis and regulation of antibacterial metabolites, competitive interactions, induction of systemic resistance in plants, and promotion of plant growth.

*Paenibacillus* is a representative strain in the field of biological control, attracting considerable attention due to its capacity to synthesize a diverse array of antibacterial active substances [14] (Figure 1). Among these, antibacterial lipopeptides serve as their primary antimicrobial agents, exemplified by streptogramin, staphylogrin, and streptozolgrin [15]. Streptogramin possesses a unique cyclic peptide structure that enables it to bind to lipopolysaccharides in the outer membrane of Gram-negative bacteria, thereby disrupting membrane integrity, increasing permeability, and ultimately causing leakage of intracellular contents and cell death [16]. Staphylogrin, while structurally analogous, similarly exerts bactericidal activity through targeting the cell membrane, thereby enhancing inhibition against Gram-negative pathogens [16]. Streptozolgrin, characterized by its cyclic lipopeptide architecture, is biosynthesized via the coordinated action of multiple enzymes encoded by a dedicated gene cluster, exhibiting broad-spectrum activity against both bacteria and fungi [16]. These not only directly compromise cellular membranes but also interfere with cell wall biosynthesis and inhibit protein synthesis, collectively impairing key physiological processes in pathogenic microorganisms [17].

In addition, *Paenibacillus* produces other classes of antibacterial metabolites. Polyketide compounds, as significant secondary metabolites, feature complex polycyclic frameworks and exhibit a wide range of biochemical activities, affording the organism versatile chemical defense mechanisms in microbial competition [18]. Volatile organic compounds (VOCs), owing to their high volatility and diffusivity, represent an effective and non-contact antimicrobial strategy [19]. In addition to direct suppression of pathogen growth, VOCs have been demonstrated to activate plant defense signaling pathways, contributing to the induction of systemic resistance and offering enhanced protection to host plants [20]. Collectively, these metabolites—through distinct modes of action and biosynthetic origins—form a multi-layered and integrated antibacterial defense system in *Paenibacillus*.

### 2.2. Competitive Effect

*Paenibacillus*, as an efficient biocontrol bacterium, has a competitive ability in the root-associated microecosystem is one of the important mechanisms for its biological control [21]. This bacterium not only competes with pathogenic bacteria for water and oxygen, but also has an advantage in obtaining key nutrients such as nitrogen, phosphorus, and iron [22]. Especially iron, as a necessary trace element for microbial growth, often becomes a limiting factor. *Paenibacillus* can synthesize and secrete efficient iron carriers—such as bacillibactin—to specifically chelate the trivalent iron ions in the environment. This process significantly reduces the available iron concentration for pathogenic bacteria, thereby inhibiting their growth and reproduction. It demonstrates its competitive advantage in the micro-ecological niche [23].

Meanwhile, this bacterium has the ability to rapidly colonize on the surface of plant roots and form a stable biofilm structure [24]. This biofilm not only effectively acts as a physical barrier to prevent pathogenic bacteria from invading, but also serves as the foundation for the long-term residence and functional expression of *Paenibacillus* in the rhizosphere [25]. The complex extracellular matrix network within the biofilm helps the bacteria aggregate, enhances environmental resilience, and inhibits the colonization of pathogenic bacteria through the spatial exclusion effect [26]. Therefore, the establishment of the biofilm is not only an expression of its ecological adaptability, but also a key link for it to exert a sustained biological control effect. In summary, *Paenibacillus* establishes a strong competitive barrier by comprehensively controlling nutrient resources and ecological niches, laying the foundation for its stable application in agricultural ecosystems.

### 2.3. Induction of System Resistance

The function of *Paenibacillus* in inducing plant systemic resistance (ISR) has been extensively studied and recognized [27]. It can trigger the activation of the jasmonic acid (JA) and ethylene (ET) signaling pathways within plants by secreting various microbial-associated molecular patterns (MAMPs) or metabolic signaling molecules, such as lipopeptides and volatile organic compounds (VOCs) [28]. These signaling molecules act as exogenous inducers and can “pre-activate” the plant immune system, enhancing its response speed and intensity to subsequent pathogen infection [29]. The activation of the JA/ET pathway not only strengthens local tissue defense responses but can also trigger systemic resistance enhancement throughout the plant through long-distance signal transmission [30]. This pre-established defense state enables plants to respond rapidly to pathogen attacks even in unstimulated areas, significantly improving their disease resistance. This mechanism does not rely on specific disease-resistant genes and has broad-spectrum and persistence, making it an important way for *Paenibacillus* to achieve indirect biocontrol.

### 2.4. Promote Plant Growth

In addition to having remarkable biocontrol functions, *Paenibacillus* also exhibits excellent plant-promoting characteristics [31]. It can convert atmospheric nitrogen into ammonia, a form that plants can absorb, through nitrogen fixation, thereby increasing the biological availability of soil nitrogen and directly supplementing the nutrients needed by plants [32]. At the same time, this bacterium has the ability to dissolve phosphorus, converting insoluble inorganic phosphates into soluble phosphorus, which promotes the absorption and utilization of phosphorus by plant roots [33]. Moreover, *Paenibacillus* can synthesize plant growth hormone substances, such as indole-3-acetic acid (IAA), regulating cell division and elongation processes, promoting the development of lateral roots and root hairs, and thereby enhancing the overall absorption capacity and growth potential of plants [34]. These promoting mechanisms complement the biocontrol functions, not only enhancing the plant’s own stress resistance and disease resistance, but also promoting the improvement of crop yield and quality, achieving “disease prevention + yield increase” dual ecological benefits. Thus, *Paenibacillus* has broad application prospects in sustainable agriculture and green control systems.

## 3. Genetic Editing of *Paenibacillus*

### 3.1. The Gene Editing Technology of Paenibacillus

The gene editing and functional gene knockout or overexpression of *Paenibacillus* are the core strategies in metabolic engineering modification [35]. The strategies must effectively address inherent challenges, including the robust cell wall of Gram-positive bacteria and the high dependence on homologous recombination efficiency. Currently, the temperature-sensitive suicide plasmid system is widely used due to its high flexibility. Using the pRN5101 plasmid as the carrier, which can stably replicate at 28 °C but lose replication ability at 39 °C, and combining with the chloramphenicol resistance gene as a screening marker, the precise knockout of the target gene can be achieved [36]. Charles et al. successfully knocked out the key gene *pmxE* for polypeptide synthesis using this system [37]. This not only verified the function of the gene but also enabled the construction of an engineered bacterial strain without antibiotic resistance markers. This fully demonstrates the potential of this technology in biological engineering applications [38].

The CRISPR/Cas9-derived technologies have brought revolutionary progress to the field of gene editing [39]. To address the problem of cytotoxicity often caused by the heterologous expression of Cas9 protein in *Paenibacillus*, researchers innovatively developed the CRISPRi (CRISPR interference) and CRISPRa (CRISPR activation) systems [40,41]. By forming a complex of the inactivated Cas9 (dCas9) with specific sgRNA and targeting the promoter region of the gene, precise and reversible regulation of the expression of the target gene can be achieved, which is suitable for dynamic metabolic regulation research. In addition, the mutation library technology based on mini transposons, such as high-throughput transposon insertion sequencing (Tn-seq), can achieve random gene inactivation throughout the genome and combine with phenotypic screening to accelerate the identification and analysis of functional genes [42].

The traceless gene editing technology offers an efficient and marker-free solution. By knocking out the uracil phosphoribosyltransferase gene (*upp*), a uracil nutritional-deficient host strain is constructed, and combined with 5-fluorouracil (5-FU) for reverse screening, gene editing without resistance markers can be achieved [43]. The positive clone screening rate can reach over 90%, significantly superior to traditional methods. The coordinated development of these various technologies has driven the gene editing of *Paenibacillus* towards higher precision, higher efficiency, and tracelessness, laying a solid technical foundation for its in-depth development in diversified application scenarios such as industrial fermentation, agricultural biocontrol, and synthetic biology [44].

### 3.2. Functional Gene Knockout and Metabolic Flux Redirection

*Paenibacillus* possesses a complex metabolic network, and functional gene knockout strategies should be guided by transcriptomic and metabolomic data to identify and target key rate-limiting steps [45]. This enables rational redirection of metabolic flux and enhances the synthesis efficiency of target products. The lactate dehydrogenase genes (*ldh*1 and *ldh*3) play critical roles in the 2,3-butanediol (2,3-BD) biosynthesis pathway. Studies have demonstrated that deletion of *ldh3* significantly reduces lactic acid accumulation, thereby redirecting carbon flux toward the 2,3-BD pathway and increasing product yield [46]. To prevent the formation of exopolysaccharides (EPSs) during 2,3-butanediol (2,3-BD) fermentation, the EPS biosynthetic pathway in *Paenibacillus polymyxa* was disrupted via homologous recombination. The gene encoding levansucrase, a key enzyme required for EPS production in this bacterium, was successfully inactivated. When grown on sucrose and glucose, respectively, the resultant levansucrase-deficient mutant of *P. polymyxa* produced only 2.5 ± 0.1 g/L and 1.2 ± 0.2 g/L of EPS [47].

Overexpression of the butanediol dehydrogenase gene (*bdh*) requires precise control over expression strength and timing. Introduction of a strong promoter to drive *bdh* expression has been shown to significantly enhance 2,3-BD accumulation under oxygen-limited conditions, bringing yields close to theoretical maximums [48]. Furthermore, modulation of sporulation-related genes (*spo0A* and *spoIIE*) exemplifies fine-tuned regulation of cellular physiology: restoration of *spo0A* expression promotes transmembrane sugar transport and directly improves substrate utilization efficiency, whereas deletion of *spoIIE* may accelerate cell growth but potentially suppresses secondary metabolite production, highlighting the need to balance growth and biosynthetic capacity [49].

By combining *ldh*3 knockout, *bdh* overexpression, and spo0A functional restoration, the research team successfully developed an engineered strain with high-efficiency 2,3-BD production. A crude poplar wood hydrolysate containing 27 g/L glucose and 15 g/L xylose was employed as a feedstock for the biosynthesis of 2,3-butanediol, achieving a maximum yield of 465 g/kg; that corresponds to 93% of the theoretical yield. The combined titer of 2,3-butanediol and ethanol reached 21.7 g/L under optimized conditions. This study demonstrates a novel and effective strategy for engineering *P. polymyxa* to efficiently convert unrefined lignocellulosic hydrolysates into renewable biofuels [49]. This strain exhibited robust performance in utilizing complex lignocellulosic substrates such as poplar wood hydrolysate, demonstrating efficient conversion of non-food biomass. These results not only validate the effectiveness of multi-gene metabolic engineering but also offer a promising technological framework for the biorefining of lignocellulosic feedstocks [49].

## 4. Construction of Synthetic Biology Platform for *Paenibacillus* and Improvement of Product Yield

### 4.1. Construction Strategy of the Synthetic Biology Platform

In synthetic biology research, genome minimization and reduction in metabolic burden represent fundamental strategies for optimizing the synthetic biology platform of *Paenibacillus* [50]. Through systematic deletion of non-essential genes—such as those within secondary metabolite biosynthesis gene clusters—the strain’s limited metabolic resources can be effectively reallocated [51]. This approach not only significantly enhances cell growth rate but also improves glucose utilization efficiency, thereby illustrating the intrinsic interplay between metabolic homeostasis and resource distribution [52]. Furthermore, the construction of promoter libraries coupled with rational optimization of ribosome binding sites (RBSs) enables multilayered control over gene expression [53]. By employing promoters and RBS variants of varying strengths, precise tuning of target gene expression levels is achievable [54]. For instance, driving *alsS* expression with the strong P43 promoter markedly increases the synthesis rate of acetoin (acetyl lactate) [55]. These regulatory strategies underscore both the precision of genetic control and the inherent complexity and adaptability of microbial systems. Although Bacillus subtilis possesses a naturally efficient protein secretion system, extracellular proteases produced by the wild-type strain often compromise the stability of heterologous products. Targeted knockout of key protease-encoding genes, including *aprE* and *nprE*, substantially improves the integrity of secreted proteins. This genetic intervention not only mitigates product degradation but also leads to a notable increase in 2,3-butanediol yield [56]. The synergistic integration of these strategies has enabled the development of a robust and high-performance synthetic biology platform, establishing a solid foundation for the expanded application of *Paenibacillus* in industrial biotechnology and facilitating its transition from traditional biocontrol functions to the production of high-value biochemicals.

### 4.2. Design of Dynamic Regulation Systems

The design of dynamic regulation systems plays a pivotal role in achieving efficient metabolic flux allocation during the development of the *Paenibacillus* synthetic biology platform [57]. The incorporation of quorum-sensing circuits allows for the establishment of feedback-regulated control loops responsive to intracellular metabolite concentrations. For example, the LuxI/LuxR system derived from *Escherichia coli* activates *bdh* gene expression when intracellular acetyl-CoA reaches a predefined threshold, thereby preventing premature carbon flux diversion and ensuring optimal resource utilization throughout the fermentation process [58].

Oxygen-responsive regulatory switches address the impact of variable dissolved oxygen levels on metabolic pathways. The PresDE promoter, capable of sensing environmental oxygen tension, effectively drives *bdh* expression under oxygen-limited conditions, resulting in a 12% improvement in 2,3-butanediol yield compared to constitutive expression [49]. This mechanism offers a strategic approach to optimizing product formation through environmental cue-based regulation and provides a framework for dynamic control in advanced bioprocesses.

The successful implementation of CRISPR interference (CRISPRi) technology in *Paenibacillus* has introduced a powerful tool for tunable gene repression [40]. The dCas9-sgRNA complex enables specific targeting and downregulation of lactic acid pathway genes such as *ldh3*. By temporally silencing this competing pathway during mid-to-late fermentation, the purity of 2,3-butanediol rises from 92% to 98% [49]. This dynamic metabolic redirection not only enhances product fidelity but also minimizes wasteful consumption of metabolic precursors, offering a more sophisticated and adaptable paradigm for metabolic engineering. Collectively, these refined regulatory modules significantly enhance the biosynthetic capacity of *Paenibacillus*.

### 4.3. Product Diversification and Chassis Strain Enhancement

The advancement of the *Paenibacillus* synthetic biology platform has unlocked significant potential for product diversification and chassis strain optimization. The biosynthesis of high-value compounds now extends beyond conventional products like 2,3-butanediol to include heterologous production of bioactive molecules such as indole-3-acetic acid (IAA) and astaxanthin [49,59,60]. Through targeted disruption of competing phenylalanine pathway genes and heterologous expression of *Pseudomonas*-derived tryptophan 2-monooxygenase, engineered strain BD27 was developed, achieving an IAA titer of 61.55 μg/mL—a 6.1-fold increase over the wild-type strain [61]. This milestone not only broadens the metabolic capabilities of *Paenibacillus* but also presents a sustainable route for the biological production of plant growth regulators.

Concurrently, the efficient utilization of lignocellulosic hydrolysates highlights the strain’s proficiency in converting renewable feedstocks. Rational co-expression of xylose isomerase and xylulokinase enables the engineered strain to simultaneously metabolize glucose and xylose, achieving a 2,3-butanediol yield of 480 g/kg [56]. This performance provides critical technological support for biorefinery processes based on low-cost, abundant biomass sources such as poplar wood and agricultural residues.

Moreover, the development of specialized microbial agents for saline-alkaline soils demonstrates the strain’s adaptability and agronomic potential. Overexpression of osmotic stress response and biofilm-forming genes resulted in a stress-tolerant composite inoculant that, when applied to moderately saline-alkaline soil, increased tomato yield by 25% and reduced fertilizer input by 30% [62,63,64,65]. This innovative approach offers a sustainable solution for ecological restoration of degraded lands and supports the advancement of environmentally responsible agriculture through improved resource efficiency and ecosystem resilience.

## 5. Challenges and Frontier Strategies: Towards Precise Design and Efficient Application of Paenibacillus

### 5.1. Current Challenges

**Figure 2:**
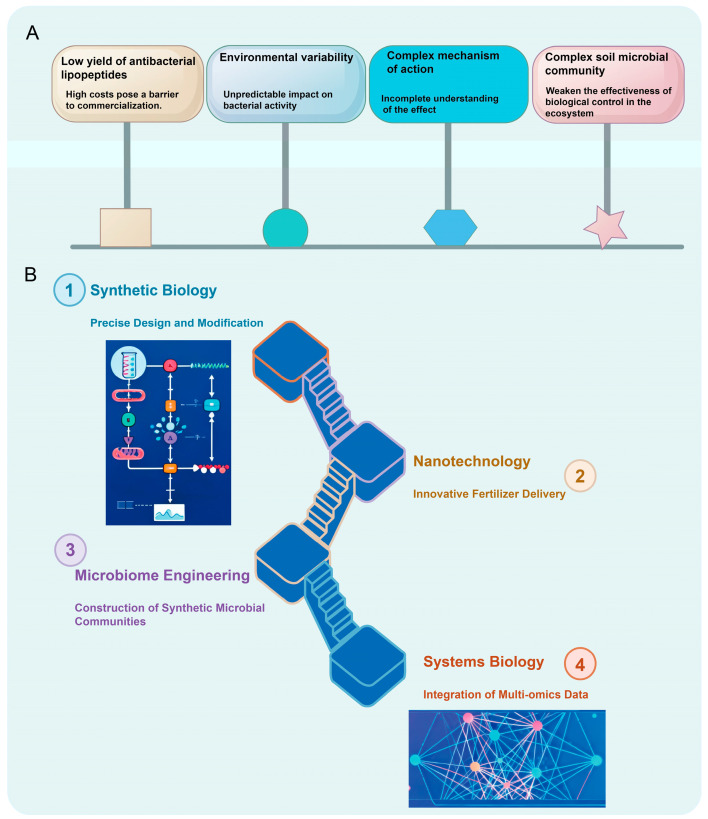
Challenges and frontier strategies in *Paenibacillus* research. (**A**) Current challenges in the applications of *Paenibacillus*. (**B**) Emerging technologies and integrated solutions for enhancing *Paenibacillus* Utilization.

The application of *Paenibacillus* as a biological control agent in modern agriculture faces multiple complex challenges (Figure 2). Field performance is often inconsistent, a phenomenon closely linked to the variability of environmental factors and the high diversity of soil microbial communities [66]. Fluctuations in environmental conditions significantly affect the survival, colonization, and functional activity of *Paenibacillus*, thereby compromising the reliability of its practical deployment [67]. Moreover, dynamic shifts in soil microbial community structure across different ecosystems may interfere with the intended function of the target strain or even suppress its biocontrol efficacy [68]. This ecological uncertainty necessitates systematic investigation to enable the rational design of strains and their effective adaptation to diverse field environments.

Low yields of antibacterial lipopeptides coupled with high production costs represent major bottlenecks hindering commercialization [69]. Although these compounds exhibit strong bioactivity against plant pathogens under laboratory conditions, this efficacy has not been successfully translated into economically scalable industrial processes [70]. There is an urgent need to enhance process efficiency through fermentation optimization, metabolic flux control, and the development and integration of novel bioreactor systems—key steps toward achieving cost-effective large-scale production and facilitating industrial adoption.

Furthermore, the mode of action of *Paenibacillus* is highly complex and multifaceted, involving intricate metabolic pathways and extensive interaction networks with other microorganisms and host plants [71]. While evidence supports its roles in pathogen inhibition, induction of systemic resistance (ISR), and plant growth promotion, the synergistic interplay and regulatory logic among these mechanisms remain poorly understood [72]. To realize predictable and high-performance applications, it is essential to systematically dissect the underlying molecular networks and establish a comprehensive framework linking mechanistic insights to field-level outcomes. This would bridge the gap between fundamental research and real-world implementation. In summary, despite its considerable promise in sustainable agriculture, the widespread adoption of *Paenibacillus* depends on the strategic integration of innovative approaches and advanced biotechnologies.

### 5.2. Cutting-Edge Technologies and Solutions

Advances in synthetic biology and metabolic engineering have provided powerful platforms for the rational redesign and functional enhancement of *Paenibacillus*. Leveraging precise gene editing tools such as CRISPR-Cas systems, researchers can fine-tune key metabolic pathways, optimize carbon flux allocation, and substantially improve the biosynthesis of target compounds—including antimicrobial lipopeptides [39]. A particularly promising frontier is the development of “intelligent” engineered strains capable of sensing specific pathogen-associated signals or environmental cues (e.g., pH, temperature, quorum-sensing molecules), and autonomously activating defense-related gene expression [73]. Such stimuli-responsive systems enable context-dependent responses and spatiotemporally controlled product release, greatly enhancing the precision and resource efficiency of biological control.

The emergence of microbiome engineering has transcended the limitations of single-strain applications by positioning *Paenibacillus* as a central component within synthetic microbial consortia. Through rational assembly of functionally complementary partner species, these engineered communities enhance the root- and leaf-associated colonization capacity of the target strain while improving its ecological resilience and functional persistence in heterogeneous soil environments [74]. These designed consortia function as modular, self-regulating ecosystems that deliver synergistic benefits in crop-microbe interactions, offering robust and adaptable solutions for sustainable agricultural practices [75].

Systems biology and multi-omics integration are now pivotal in deciphering the complex physiological and functional landscape of *Paenibacillus* [76]. By combining genomic, transcriptomic, proteomic, and metabolomic datasets, researchers can reconstruct genotype-to-phenotype mapping networks, enabling a holistic understanding of metabolic regulation, stress response mechanisms, and host-microbe crosstalk [77]. This integrative approach facilitates the transition from phenomenological observation to predictive modeling, providing data-driven strategies for strain improvement and process optimization.

Concurrently, innovations in nanotechnology and formulation science are revolutionizing the delivery and stability of microbial inoculants [78]. Advanced delivery platforms—such as nanoencapsulation, microencapsulation, and hydrogel-based carriers—effectively shield bacterial cells from environmental stressors, including UV radiation, desiccation, and extreme pH [79]. These systems significantly extend shelf life and improve field viability, ensuring higher cell survival upon application. Beyond protection, they enable controlled and targeted release of active agents, thereby expanding the applicability of microbial products in precision agriculture [80]. Notably, however, the final example referencing *Lactobacillus plantarum* appears misplaced in this context and should be corrected to maintain taxonomic consistency with the focus on *Paenibacillus*.

## 6. Conclusions

*Paenibacillus* exhibits significant and diverse application potential across agriculture, medicine, and industrial biotechnology. In agriculture, it functions as a potent biological control agent, enhancing crop productivity and plant stress tolerance through effective suppression of pathogens and improvement of soil health. In the medical field, this bacterium serves as a promising source for novel antibiotic discovery, offering innovative strategies to combat the growing threat of antimicrobial resistance. In industrial applications, its robust enzymatic production capabilities and versatile metabolic pathways have contributed substantially to the advancement of green chemistry, demonstrating considerable commercial and environmental promise. Future research is expected to focus on precision microbial engineering, enabling the construction of functionally defined and controllable engineered strains for the efficient execution of targeted biological processes. Concurrently, in-depth investigation into ecological interaction networks is essential to elucidate the complex relationships between *Paenibacillus*, plants, and other microorganisms at the microbiome level, uncovering mechanisms of functional synergy and competition that underpin ecosystem stability and service provision. Interdisciplinary integration will be a key driver of future breakthroughs, with synergistic advances in materials science, artificial intelligence, and bioinformatics significantly enhancing the accuracy of gene cluster functional annotation and the optimization of fermentation processes. Through cross-disciplinary collaboration and technological convergence, research on *Paenibacillus* is poised to enter a new era, positioning it as a critical biological resource in addressing global challenges in sustainable agriculture and public health.

## Data Availability

No new data were created or analyzed in this study. Data sharing is not applicable to this article.
